# Research trend of microbiota-gut-brain axis in Alzheimer’s disease based on CiteSpace (2012–2021): A bibliometrics analysis of 608 articles

**DOI:** 10.3389/fnagi.2022.1036120

**Published:** 2022-11-22

**Authors:** Zi-Long Li, Hao-Tian Ma, Meng Wang, Yi-Hua Qian

**Affiliations:** ^1^Department of Human Anatomy and Histoembryology, School of Basic Medical Sciences, Xi’an Jiaotong University, Xi’an, Shaanxi, China; ^2^Institute of Neuroscience, Translational Medicine Institute, Xi’an Jiaotong University Health Science Center, Xi’an, Shaanxi, China; ^3^College of Forensic Science, Xi’an Jiaotong University, Xi’an, Shaanxi, China

**Keywords:** microbiota-gut-brain axis, microbiota, Alzheimer’s disease, research trends, bibliometric analysis, Web of Science database, CiteSpace

## Abstract

**Background:**

Recently, research on the microbiota-gut-brain axis (MGBA) has received increasing attention, and the number of studies related to Alzheimer’s disease (AD) has increased rapidly, but there is currently a lack of summary of MGBA in AD.

**Objective:**

To capture research hotspots, grasp the context of disciplinary research, and explore future research development directions.

**Methods:**

In the core dataset of Web of Science, documents are searched according to specific subject words. CiteSpace software is used to perform statistical analysis on measurement indicators such as the number of published papers, publishing countries, institutions, subject areas, authors, cocited journals, and keywords, and to visualize of a network of relevant content elements.

**Results:**

The research of MGBA in AD has shown an upward trend year by year, and the cooperation between countries is relatively close, and mainly involves the intersection of neuroscience, pharmacy, and microbiology. This research focuses on the relationship between MGBA and AD symptoms. Keyword hotspots are closely related to new technologies. Alzheimer’s disease, anterior cingulate cortex, inflammatory degeneration, dysbiosis, and other research are the focus of this field.

**Conclusion:**

The study revealed that the research and development of MGBA in AD rapidly progressed, but no breakthrough has been made in the past decade, it still needs to be closely combined with multidisciplinary technology to grasp the frontier hotspots. Countries should further strengthen cooperation, improve the disciplinary system, and increase the proportion of empirical research in all research.

## Introduction

The etiological mechanism of Alzheimer’s disease (AD) is complex, and can lead to a series of symptoms such as severe dementia, and its prognosis is poor. Although some targeted drugs have been marketed, there is currently no complete cure, and only limited methods can be used for short-term symptomatic treatment (Song et al., 2021; Srivastava et al., 2021; Stoiljkovic et al., 2021). The other main concept of the microbiota-gut-brain axis (MGBA) refers to the effect of microorganisms living in the gut or on the enteric nerve and vagus nervous system through the microbial structural components, short-chain fatty acids, and neuroactive molecules, in particular the gut microbiota itself have been demonstrated to be involved not only in brain development, but also in behavior regulation, neuropsychiatric disorders, and neurodegenerative diseases including AD (Escobar et al., 2022). Research on MGBA and AD has been increasing in the past decade. Most of the literature revealed that patients with AD, but not those with mild cognitive impairment, demonstrated significantly reduced GM diversity compared to healthy older adults (Hung et al., 2022). A growing body of literature indicates that gut microbial composition is altered in AD and that therapeutic interventions incorporating the microbiome may be a viable target for protecting against or mitigating the effects of AD (Murray et al., 2022).

Studies have reported that the gut microbiota is altered in AD. When AD patients were compared with healthy controls, AD patients exhibited diverse microbiota, an increased abundance of Bacteroidetes, and a reduced abundance of Firmicutes, Proteobacteria, and Actinobacteria, in China (Zhang et al., 2022). In America, researchers identified phylum- through genus-wide differences in bacterial abundance including decreased Firmicutes, increased Bacteroidetes, and decreased Bifidobacterium in the microbiome of AD participants (Vogt et al., 2017). Similarly, in several different kinds of AD model mice, such as SAPM8 and APP/PS1 mice, researchers also found changes in gut microbiota abundance (Bauerl et al., 2018; Marizzoni et al., 2020), and mice with different fecal microbiota transplants also showed behavioral and pathological changes compared to controls (Harach et al., 2017; Fujii et al., 2019; Kim et al., 2020). Further research on the connection between MGBA and AD is necessary, and targeting and regulating specific gut microbes may be a potential treatment for AD. This paper aims to summarize the research related to MGBA and AD in the past 10 years, which will help to analyze and grasp the research situation of global MGBA in AD and provide a reference for future research on the pathological mechanism and treatment of AD, to complement the insufficiency of research in this field.

Therefore, this study applied a bibliometric strategy, based on the literature sources in the authoritative database Web of Science Core Collection, and collected all relevant research including MGBA in AD in the past 10 years (2012–2021, 0 in 2012). This paper uses CiteSpace, a visual bibliometric software [CiteSpace is designed to provide such an alternative so that we can use our datasets to answer questions about an ever-changing knowledge domain (Chen, 2016)], This paper uses CiteSpace, a visual bibliometric software, to analyze the development trend of global research, and summarizes the growth of the number of articles published in AD research, authors, institutions, countries, and research hotspots for MGBA. The differences provide references for topic selection, cooperation, and future development trends in this field.

## Materials and methods

### Literature data sources and inclusion

Literature data were collected from the Science Citation Index (SCI) of the database Web of Science Core Dataset. For the sake of comprehensiveness, Mesh Terms identifies the search subject term (Supplementary Document 1).

**Figure 1:**
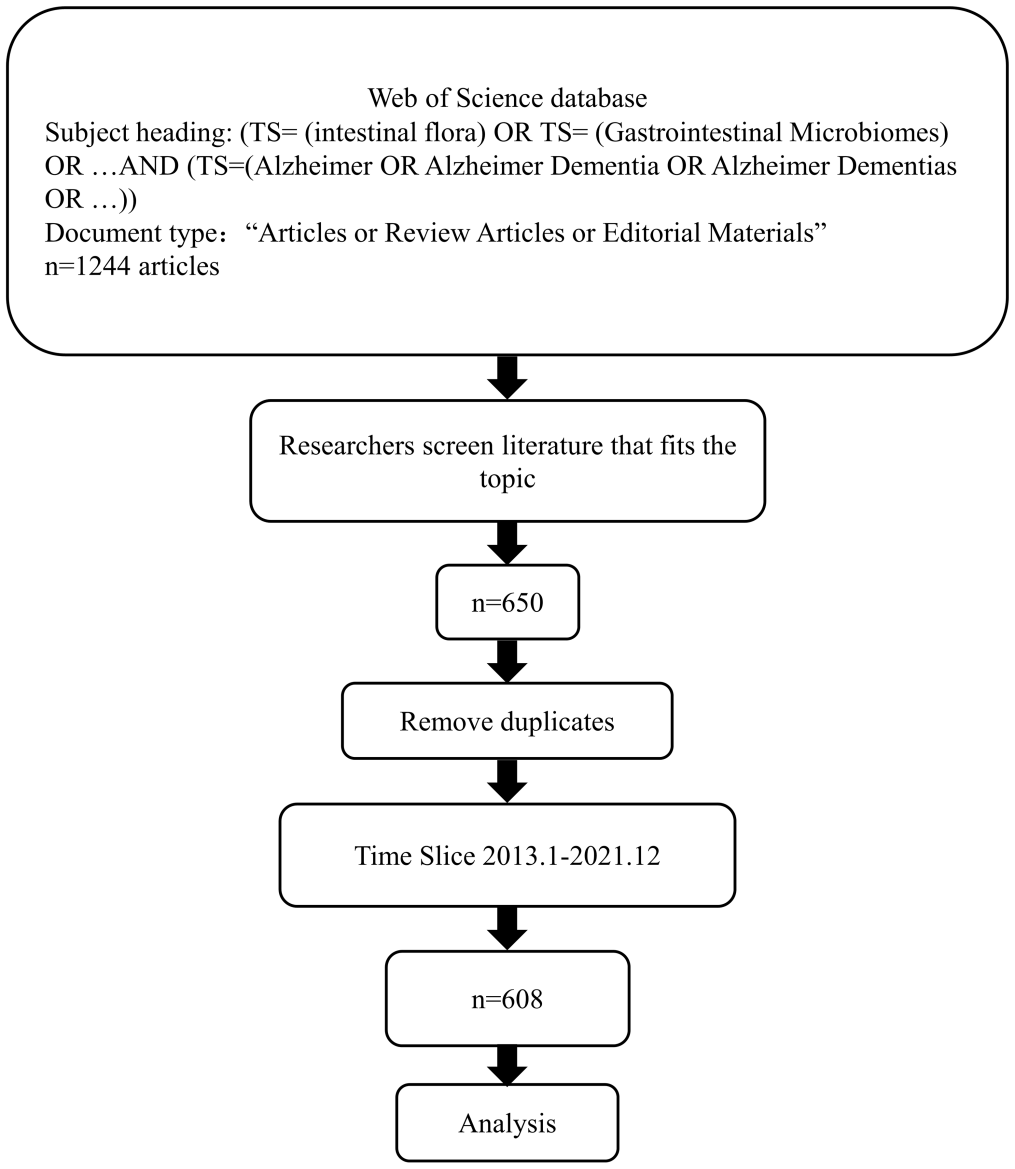
Search strategy in this study.

After retrieving the literature according to the established subject heading requirements, browsing the full text, and adding the papers that met the requirements of MGBA and AD fields to the Marked list, the record content was full record and cited references. After screening all the papers, a total of 650 papers were included, the export operation was performed, and the target file was saved in “.txt” format. The analysis materials were imported into CiteSpace.5.7.R2, and the literature was deduplicated through the software deduplication function. Since no relevant articles were published in 2012, the analysis was limited to the period from January 1, 2013, to December 31, 2021. The retrieval strategy process is shown in Figure 1.

### Quantitative analysis methods

The line graph of the annual publication volume data is drawn by GraphPad Prism (v8.0.2(263)), from January 2012 to December 2021 (the number of published papers in 2012 is 0). For quantitative analysis, the time slice was set from January 2013 to December 2021, and analyze by category. When analyzed keywords, the time slice is 1 year. The filtering strategy is TopN = 25 for the most frequently cited items, and the relationship strength algorithm is unified. For the Cosine method. When analyzing countries, institutions, authors, cited journals, and categories, the time slice is 2 years, TopN% = 100% is selected, and the screening strategy is selected as “selection criteria.” In the country (region) network analysis, it is judged whether it is a key node according to the centrality (>0.1) calculated automatically by the software. Determine the top 16 countries (regions) by the number of published documents (the two are tied for 15th) and the institutions with the top 12 by the number of published documents (the three are tied for tenth). The “Author” and “Cited author” nodes were selected to build author and author cocitation networks and identify the top 10 authors. Change the node type to “Cited Journal” to build a journal cocitation network, take the top 15 journals with “Counts>85,” and check the journal impact factor through “Journal Citation Reports” in Web of Science “Products.” Switch the node to build a subject (field) network relationship graph for “category.” Switch the “Keyword” node to implement keyword analysis. After merging synonyms, we run again to perform keyword clustering analysis. Based on the modularity of the graph information (Q value, >0.3) and the graph outline coefficient (S value, >0.5), the keyword clustering effect is evaluated, and the top terms are output. Based on the keyword clustering analysis, a timeline graph is constructed, and the keyword emergence analysis results are further obtained through “Frequency Burst History.” The threshold setting of burst detection is the default.

### Data cleaning

The authors, institutions, categories, and keywords are all cleaned, but the data that are similar but cannot be determined to be the same expression are not cleaned. For example, in category analysis, “Geriatrics & Gerontology” and “GERIATRICS & GERONTOLOGY” are cleaned as “Geriatrics & Gerontology,” and “NEUROSCIENCES & NEUROLOGY” and “Neurosciences” are cleaned as “NEUROSCIENCES & NEUROLOGY.” In the keyword analysis, the different dementia types were unified as “dementia,” the different expressions of “amyloid” were unified as “a beta,” and the different expressions of MGBA were unified as “gut-brain axi.”

## Results

### An overview of MGBA in AD

#### Number of published research papers

**Figure 2:**
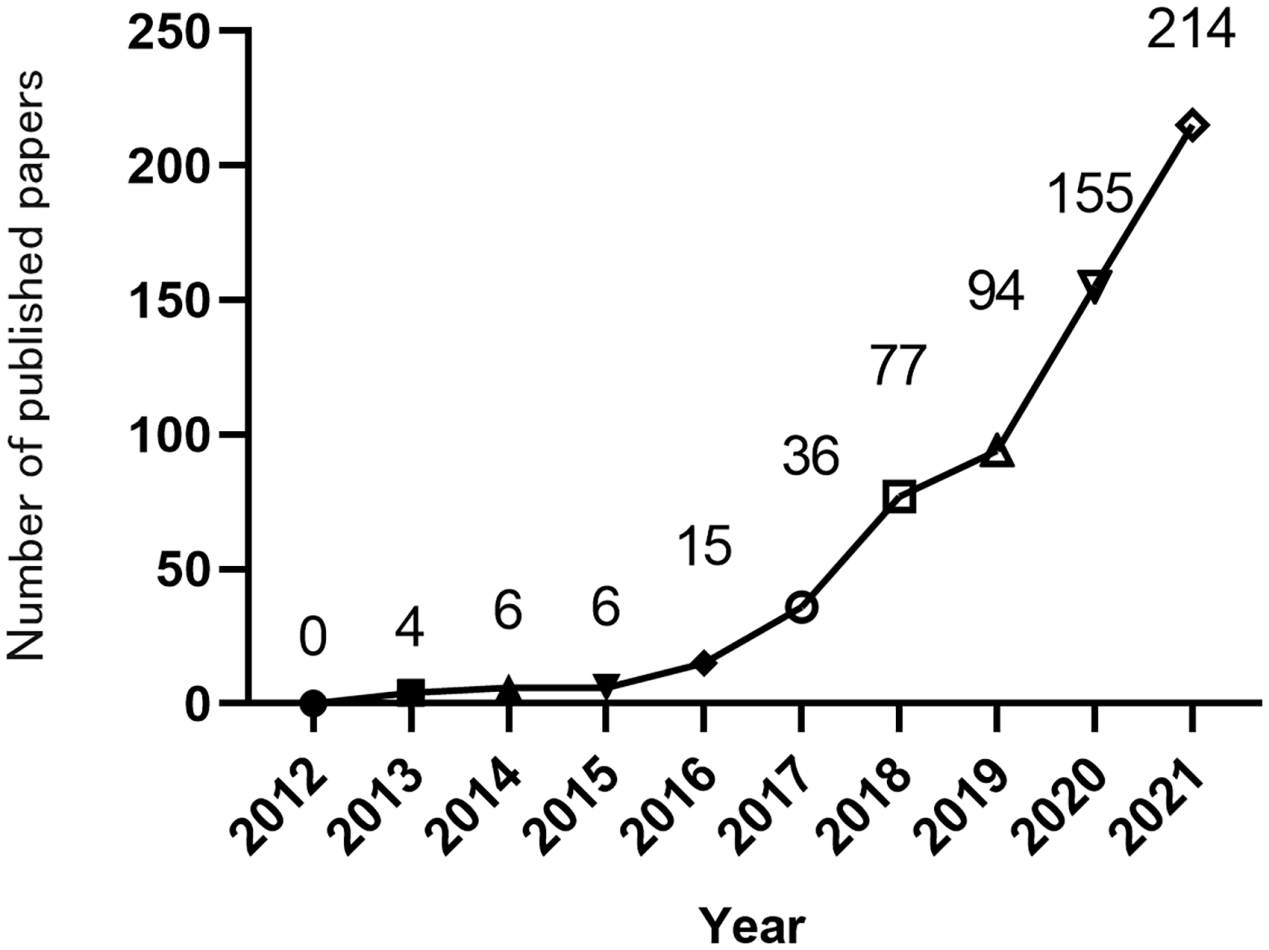
Trends of the number of published articles on MGBA in AD from 2012 to 2021.

The annual number of publications can reflect the attention of the field. Total of 608 papers in the Web of Science database in the past 10 years met the search requirements for this article. Overall, since 2012, the annual publication volume of MGBA research in AD has generally increased, as shown in Figure 2. According to the division of each year, this study divides the year information into 9 intervals. In 2012, no related articles were published. From 2012 to 2015, the related articles were published in single digits. Since 2016, the number of related articles published continued to increase. Then, the growth rate slowed down from 2018 to 2019, and the number of articles increased rapidly from 2019 to 2020, reaching 155 in 2020 and 215 in 2021.

#### Visual analysis of research countries (regions) and institutions

**Figure 3:**
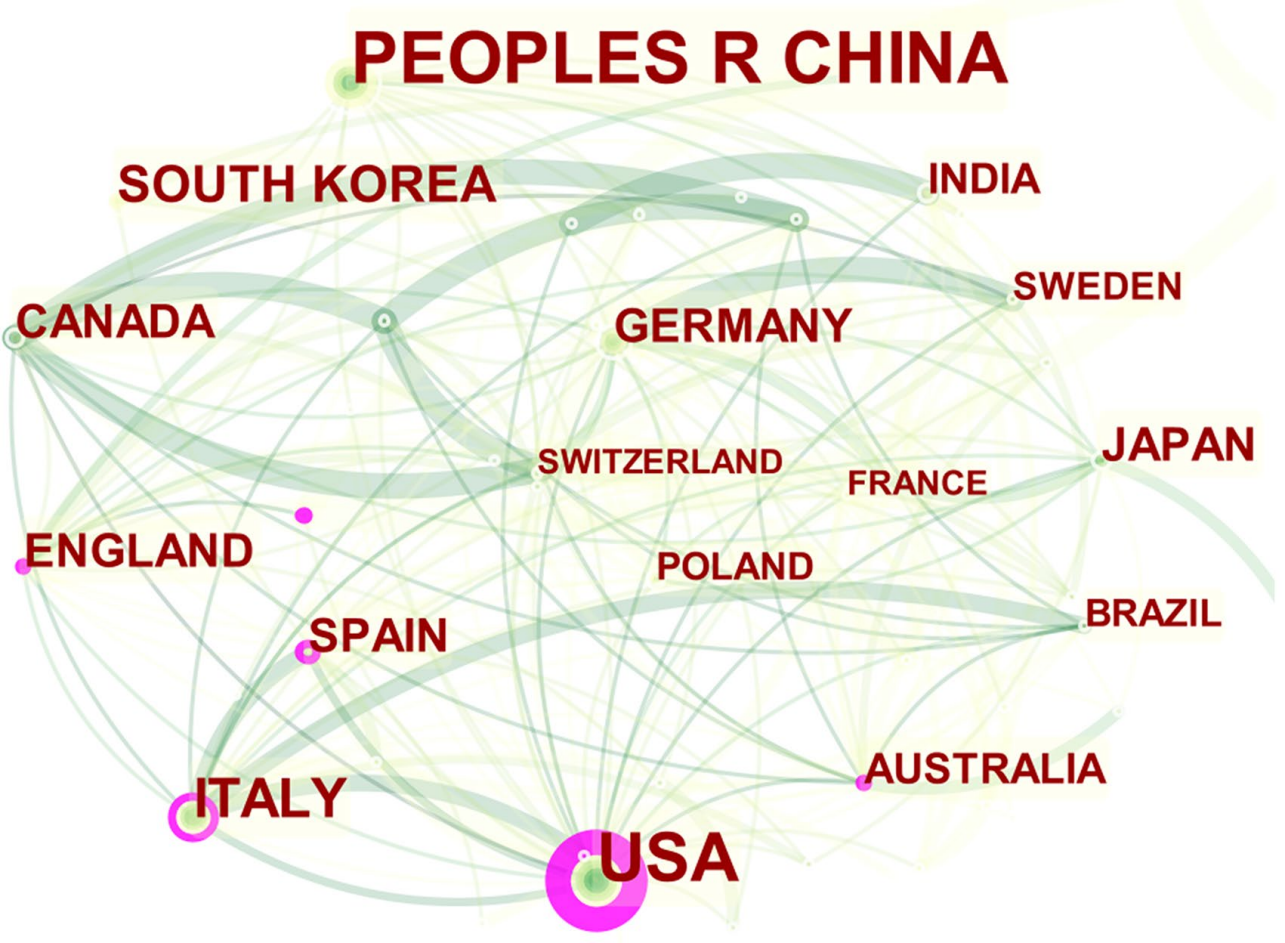
The collaboration network of major countries on MGBA in AD. The size of the nodes in the graph represents the number of publications in the country/region, and the thickness of the lines between nodes represents the strength of the relationship. The outer circle of a node is marked in purple, which means that the center degree of the node is >0.1, and the different colors of the inner circle and the connection line represent different years (for instance, dark cyan represents 2013–2014, green represents 2017–2018, and pale yellow represents 2021).

Change the Node Type in CiteSpace to “country” to visualize the national collaboration network to analyze the collaboration network of each country in the study of MGBA in AD. The visualized network diagram is shown in Figure 3. The network has a total of 59 nodes, 226 links, and a density of 0.1321, which indicates that there are many countries (regions) involved in the study of MGBA in AD, and the international cooperation relationship is close. The central nodes of the cooperation network are the United States, Italy, and other countries whose own publication volume is high and it cooperates closely with other countries. The United States has published a total of 167 articles, accounting for 27.47% of the total, with a centrality of 0.53 (>0.1), which indicates that the United States has greater influence. In contrast, the highest number of published papers in China is 184, accounting for 30.26% of the total number of papers, but the centrality is only 0.06 (<0.1). 0.03, 0.08, it can be seen that these are not key nodes.

**Figure 4:**
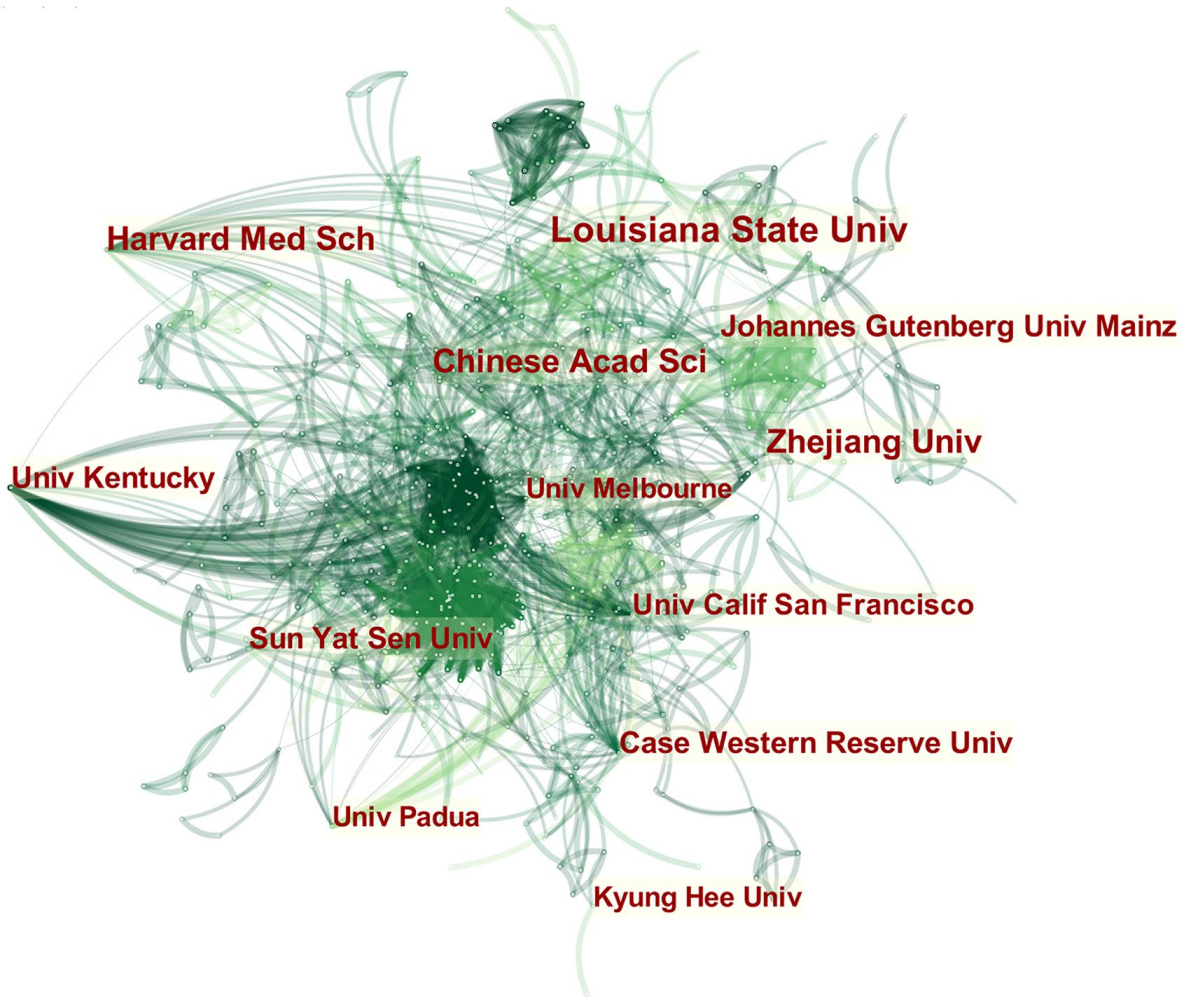
The collaboration network of top 12 institutions on MGBA in AD. The size of the nodes in the graph represents the number of publications of the institution, and the thickness of the lines between nodes represents the strength of the relationship. The different colors of nodes and connection lines represent different years (for instance, purple represents 2013–2014, blue-green represents 2017–2018, and yellow represents 2021).

By changing the node type to “institution” and constructing a global institutional interaction network (Figure 4), a total of 800 nodes and 2,667 links were obtained, indicating that there are many institutions conducting research on MGBA in AD. Among the institutions, Louisiana State Univ has the highest number of publications with 16 papers, and Chinese Acad Sci, Harvard Med Sch, and Zhejiang Univ are tied for second place with 13 papers. As shown in Table 1, the top 12 institutions in the world (in descending order of the number of issued documents), account for more than 20% of the world’s total issued documents. It can be seen that the institutions that publish papers on AD research in MGBA are mainly universities and research institutes.

**Table 1:** Top 12 institutions with maximum publications.

No.	Institution	Publications
1	Louisiana State Univ	16
2	Chinese Acad Sci	13
3	Harvard Med Sch	13
4	Zhejiang Univ	13
5	Case Western Reserve Univ	10
6	Johannes Gutenberg Univ Mainz	10
7	Sun Yat Sen Univ	10
8	Univ Kentucky	10
9	Univ Calif San Francisco	9
10	Kyung Hee Univ	8
11	Univ Melbourne	8
12	Univ Padua	8

#### Analysis of principal investigators and co-cited journals

**Figure 5:**
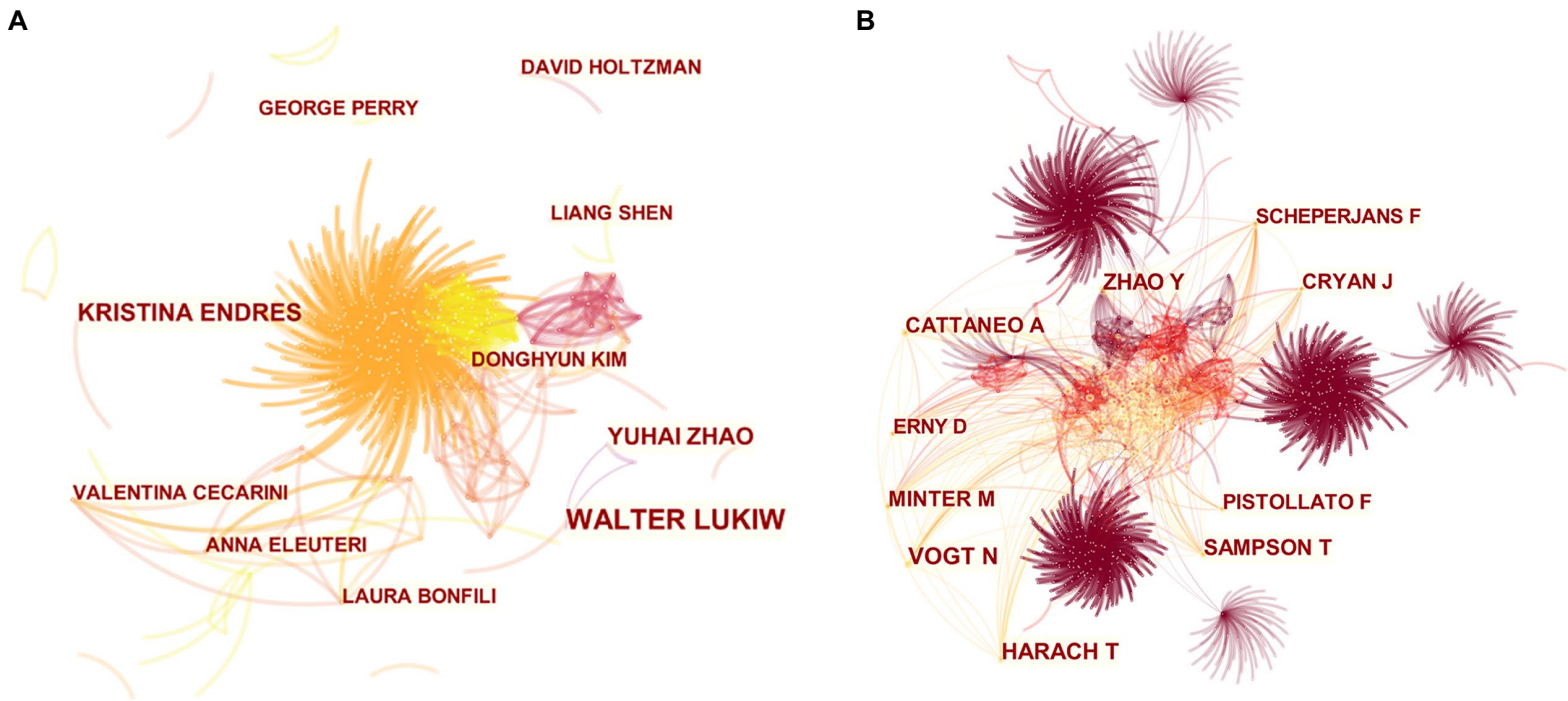
Collaboration and co-cited networks of authors. **(A)** Network of author interaction. **(B)** Network of author co-citation. The size of the nodes in the graph represents the number of publications of the institution, and the thickness of the lines between nodes represents the strength of the relationship. The different colors of nodes and connection lines represent different years (for instance, fuchsia represents 2013–2014, orange red represents 2017–2018, and yellow represents 2021 in panel **A**; fuchsia represents 2013–2014, orange represents 2017–2018 in panel **B**).

The main researchers of MGBA in AD research were analyzed from the author’s cooperation network (Figure 5A) and the author’s cocitation network (Figure 5B). WALTER LUKIW (15 papers) ranked first in the number of published papers, and the difference in the total number of papers published by the other authors with more papers was smaller (Table 2). Table 3 lists the top 10 authors with the most citations for related research, among which the top three are VOGT N, HARACH T, and ZHAO Y, with a total of 81, 67, and 61 citations, respectively.

**Table 2:** Top 10 publication authors.

No.	Name	Publications
1	Walter Lukiw	15
2	Kristina Endres	10
3	Yuhai Zhao	9
4	Laura Bonfili	6
5	Liang Shen	6
6	George Perry	6
7	Anna Eleuteri	6
8	David Holtzman	6
9	Valentina Cecarini	6
10	Donghyun Kim	6

**Table 3:** Top 10 co-cited authors.

No.	Name	Co-citations
1	Vogt N	81
2	Harach T	67
3	Zhao Y	61
4	Sampson T	55
5	Cattaneo A	54
6	Minter M	52
7	Cryan J	45
8	Pistollato F	45
9	Erny D	43
10	Scheperjans F	41

**Figure 6:**
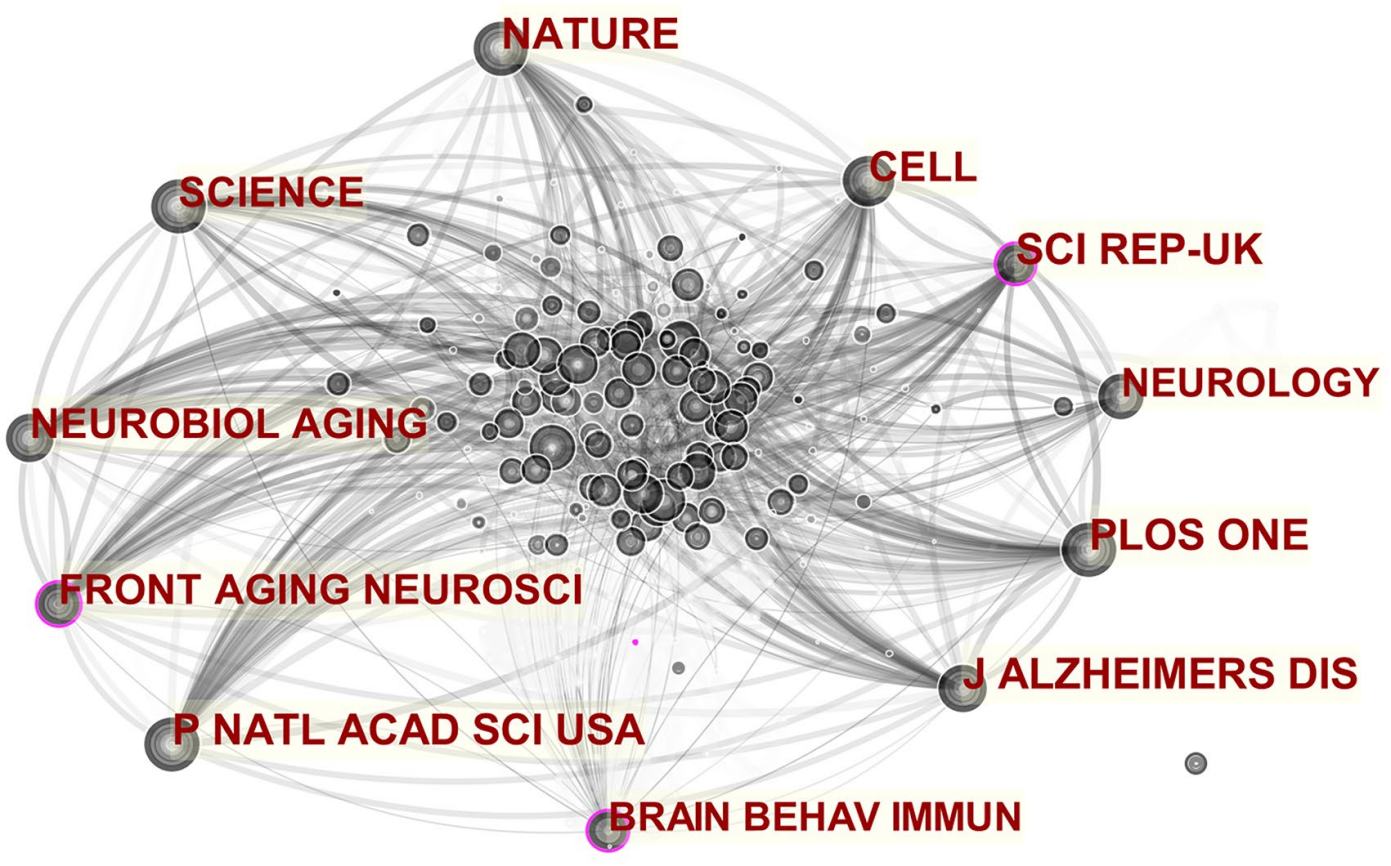
Co-cited networks of journals. The size of the nodes in the graph represents the number of publications of the institution, and the thickness of the lines between nodes represents the strength of the relationship. The different colors of nodes and connection lines represent different years (for instance, black represents 2013–2014, and light gray represents 2019–2020).

A journal cocitation analysis network was constructed to illustrate the well-known journals of MGBA in AD research (Figure 6). In the established cocitation network, the three journals “J ALZHEIMERS DIS,” “PLOS ONE” and “P NATL ACAD SCI USA” ranked the top three, with impact factors of 4.472, 3.240, and 10.700 respectively, all of which are included in the Science Citation Index A well-known journal in neuroscience, which shows that MGBA in AD research is highly valued by high-level international journals.

#### Analysis of MGBA in the subject area of AD research

**Figure 7:**
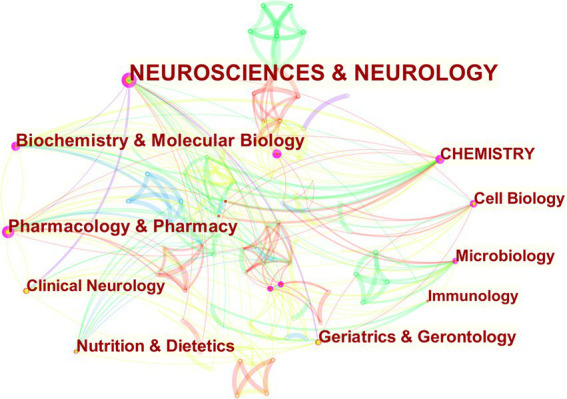
The visualization of co-occurring subject categories for MGBA in AD. The size of the nodes in the graph represents the number of publications of the institution, and the thickness of the lines between nodes represents the strength of the relationship. The different colors of nodes and connection lines represent different years (for instance, purple represents 2013–2014, green represents 2017–2018, and red represents 2021).

The study of MGBA in AD not only involves the molecular mechanism of the disease but also involves a combination of multiple fields. After selecting the node type as the “category,” a visual map of subject area co-occurrence can be obtained (Figure 7). There are 103 nodes, 352 links, and a density of 0.067, which indicates that the research on MGBA in AD involves a wide range of fields. The cross-penetration between disciplines is very significant. After removing the duplicate nodes, it can be seen from the network diagram that the research on MGBA in AD mainly involves neuroscience & neurology, pharmacology & pharmacy, biochemistry & molecular biology, geriatrics & gerontology, nutrition & dietetics, cell biology, chemistry, clinical neurology, microbiology, and immunology. The intersection of neuroscience, pharmacy, geriatrics, nutrition, chemistry, microbiology, and immunology is an important feature of research in this field. The progress of molecular biology theory and technology has greatly promoted the study of AD mechanisms.

### Analysis of research hotspots of MGBA in AD

#### Research hotspots of MGBA in AD

**Figure 8:**
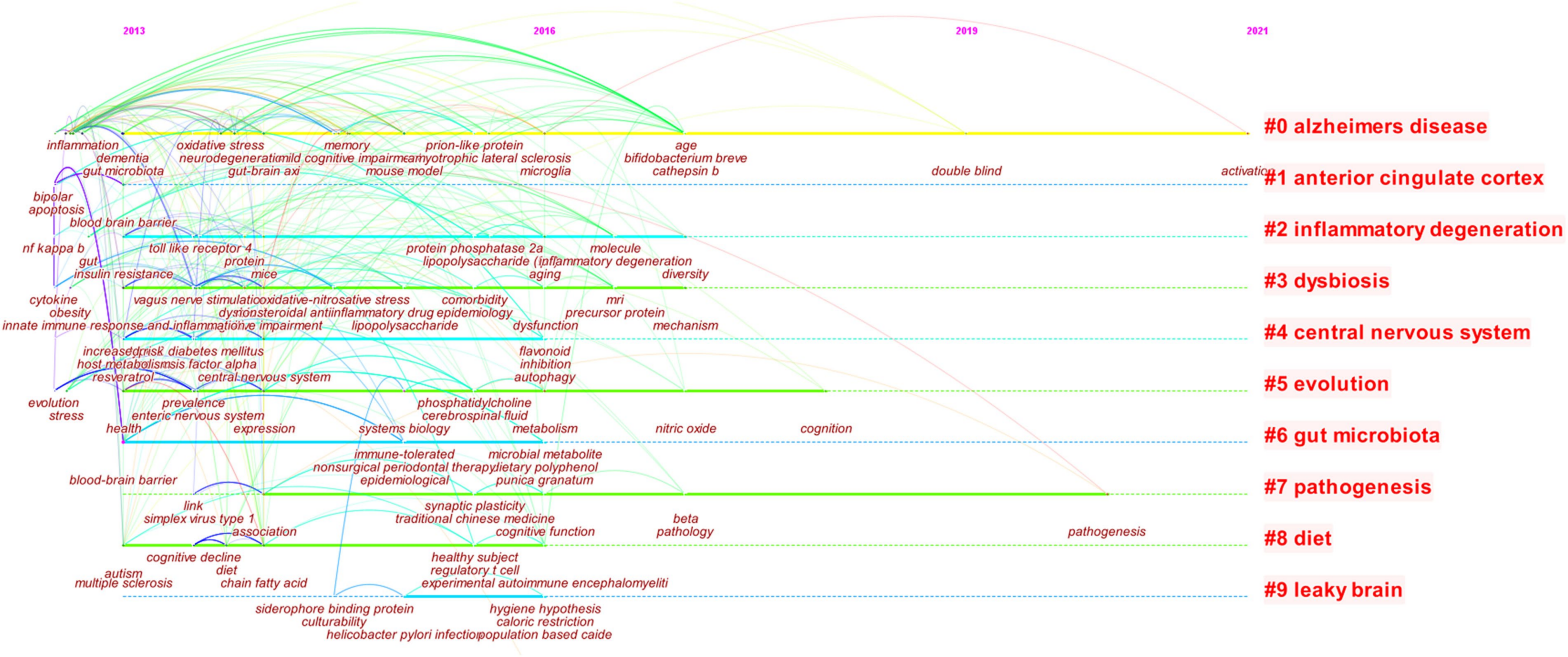
The timeline view of keywords.

Change the node type to “keyword” to build a keyword co-occurrence network, and then build a keyword clustering graph based on the co-occurrence network, with TopN = 25 (Figure 8). In cluster analysis, the larger the number of links, the Q value, and the S value, the better the clustering effect. A total of 416 nodes and 1,643 links were obtained in this clustering. The Q value was 0.7521 (*Q* > 0.3) and *S* = 0.9034 (*S* > 0.5). The results show that the clustering model is significant and can be used for analysis (Zhao et al., 2017). The clustering of keywords studied in AD by MGBA is shown in Table 4. In the table, the size of the left sequence number is negatively correlated with the popularity of the keyword.

**Table 4:** Top 10 clusters of keywords.

Cluster ID	Keywords cluster	Keywords (Top 5)
#0	Alzheimer’s disease	Alzheimer’s disease; gut microbiota; mild cognitive impairment; microbiota; neuroinflammation
#1	anterior cingulate cortex	anterior cingulate cortex; major depressive disorder; glia; fatigue syndrome; immune cells
#2	inflammatory degeneration	inflammatory degeneration; aging; neuroinflammation; reactive nitrogen species; microrna
#3	dysbiosis	dysbiosis; irritable bowel syndrome; dysfunction; mri; mild cognitive impairment
#4	central nervous system	central nervous system; type 2 diabetes mellitus; peripheral circulatory system; flavonoids; genetics;
#5	evolution	evolution; gut microbiota; neurological disorders; neuroinflammation; systems biology
#6	gut microbiota	gut microbiota; ellagitannins; pomegranate; oral; polymorphism
#7	pathogenesis	pathogenesis; helicobacter pylori; association; gastrointestinal microbiota; transcription factor
#8	diet	diet; cns diseases; inflammation; neurological disorders; multiple sclerosis
#9	leaky brain	leaky brain; hygiene hypothesis; autopoiesis; dormancy; culturability

Since 2013, the keyword clustering labels of this study mainly include the most important Alzheimer’s disease, gut microbiota, and human body structure names central nervous system, anterior cingulate cortex, pathological changes related to inflammatory degeneration, dysbiosis, pathogenesis, and others such as evolution, diet, leaky brain. Each cluster contains a lot of detailed keyword information, which can reflect the research details of the hotspot. For example, keywords such as anterior cingulate cortex, major depressive disorder, glia, fatigue syndrome, immune cells, etc. are under the anterior cingulate cortex cluster label, and dysbiosis, irritable bowel syndrome, dysfunction, MRI, mild cognitive impairment, etc. are included in the dysbiosis cluster label.

Due to the continuous development of this research field, its hotspots will continue to change over time. Simply relying on keyword clustering analysis has great limitations. Keyword clustering analysis needs to be combined with time scales to more accurately analyze the frontiers of this field.

#### The research frontier of MGBA in AD

Keyword clusters can be further visualized and analyzed with the Timeline graph. Figure 8 shows popular hotspots during the study of MGBA in AD. In 2013, the keywords of MGBA research in AD mainly focused on gut microbiota, dementia, inflammation, amyloid beta, Parkinson’s disease, probiotics, etc., mouse model, gut-brain axi, chain fatty acid, oxidative stress in 2013–2015, neurodegeneration, cognitive impairment research gradually increased, 2015–2017, microglia, metabolism, mechanism, etc. became new hotspots, 2017–2021, double-blind, pathogenesis, activation, etc., fewer hotspots.

**Figure 9:**
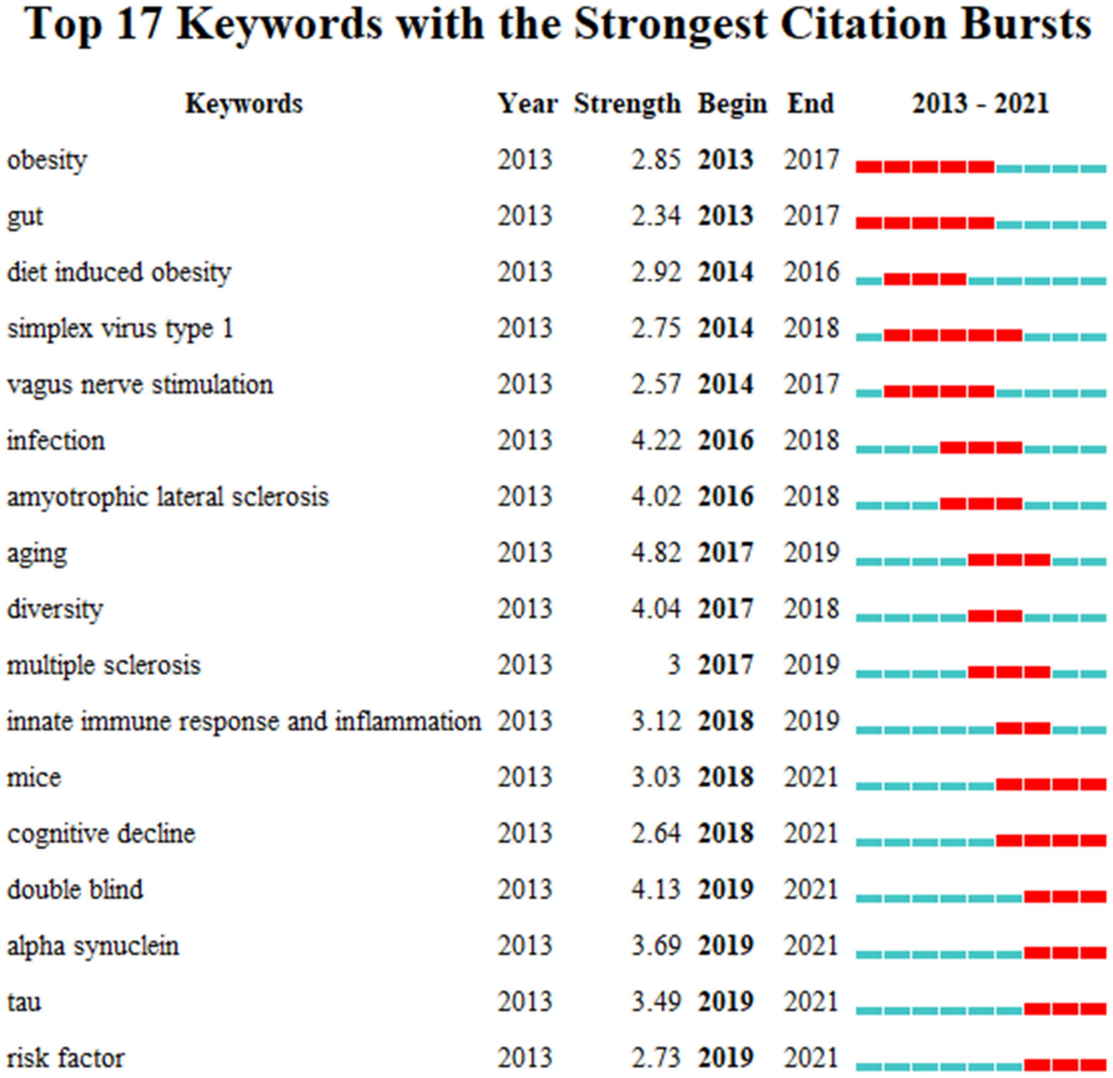
Keywords with bursts from 2013 to 2021.

Keyword bursts analysis can directly reflect the development trend of MGBA in AD research. As shown in Figure 9 from 2013 to 2017 obesity and gut had been the strongest citation bursts that is to say they have been of concern for a long time. In the middle of the decade diet-induced obesity simplex virus type 1 vagus nerve stimulation infection amyotrophic lateral sclerosis aging diversity multiple sclerosis innate immune response and inflammation were the strongest bursts. At this stage the mechanism of the disease has become a hot topic. Researchers have focused on dieting infection and the connection between the intestine and the central nervous system through peripheral nerves. From 2018 to 2021 in mice cognitive decline double-blind alpha-synuclein tau and risk factor bursts are the strongest which means that research is moving toward empirical evidence. During this time the researchers experimented with mice and the emerging alpha-synuclein and tau bursts also showed that the field is still evolving

#### Research references and co-cited authors highlighting MGBA in AD

**Figure 10:**
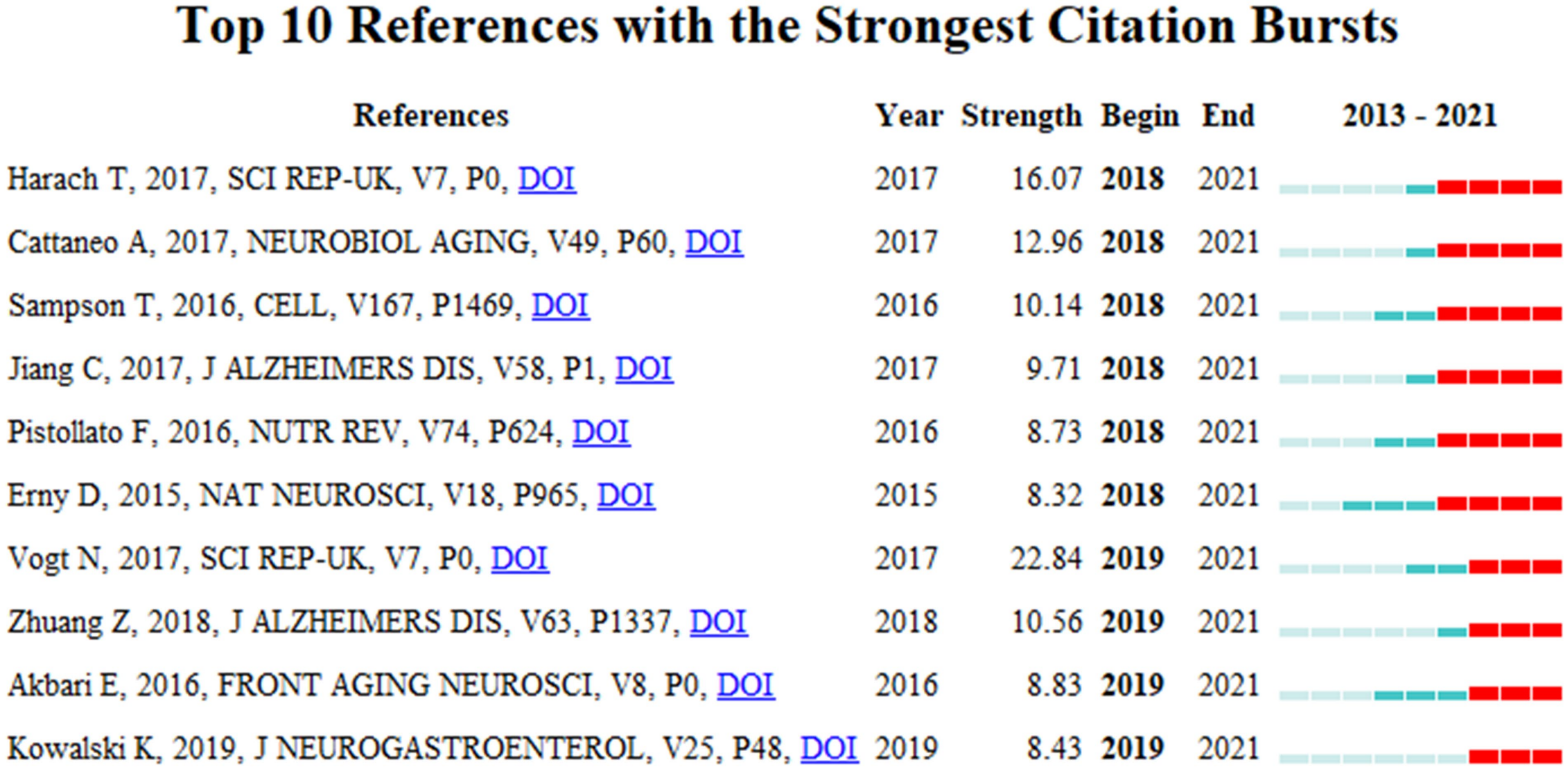
References with the strongest citation bursts from 2013 to 2021.

Articles with citation bursts show a significant increase in research interest in the field of MGBA in AD. Figure 10 shows the 10 strongest references from 2013 to 2021. The first six references received high attention from 2018 to 2021, and the last four references received high attention from 2019 to 2021, and are the focus of the current MGBA in AD research.

## Discussion

Before 2017, the number of studies related to MGBA in AD grew slowly, but in the past 5 years, the number of publications has increased rapidly, and research hotspots have been updated frequently. Therefore, it is necessary to promptly summarize and analyze the progress of this research field and the changing laws of hot spots, to help researchers maintain an international frontier vision and facilitate the development of this field. However, the current research lacks analysis of the developmental context and hotspot evolution of MGBA in AD research. Therefore, the author chose to use the visual data analysis software CiteSpace software to conduct an in-depth analysis of the development trend, hotspot evolution, countries, and institutions of MGBA research in AD.

### Country/region

The publications and citations of a country (region) can reflect its international influence and attention in the research field. Over the world, in the past 10 years, the number of published papers has shown an upward trend, and many countries in the world have participated in the research of MGBA in AD. For example, although the United States ranks second in the number of published articles, its centrality ranks first and has the greatest influence. Other examples are England, Italy, Spain, and other developed countries (regions). Although the number of publications is not very large, their centrality is >0.1, which has produced a great impetus for the development of the discipline. While Germany, France, Japan, South Korea, and other countries have published many papers, the centrality of their research is also <0.1. The author suggests that the reason may be that they prefer to use the data of their nationals for analysis (Nguyen et al., 2018; Fink et al., 2021; Gonzalez-Dominguez et al., 2021; Kim C. S. et al., 2021; Ueda et al., 2021). China, which has the largest number of published articles, has a centrality of <0.1 and limited international influence, which may be due to the inclusion of many traditional Chinese medicines (TCMs). For instance, Liuwei Dihuang decoction, baicalein, Yi-Zhi-An-Shen granules, Jatrorrhizine, etc. (Wang et al., 2016; Gao et al., 2018; Wang J. H. et al., 2019; Wang S. et al., 2019; Yue et al., 2019).

### Multidisciplinary integration is the characteristic of MGBA in AD

Based on co-occurrence category analysis, can be used in interdisciplinary research to analyze the internal relationship between different discipline categories by constructing a discipline (field) network map (Huang et al., 2020). This study found that the research design of MGBA in AD is multidisciplinary, especially in neuroscience, geriatrics, nutrition, microbiology, etc., with strong research and application value. The experimental method and sample collection have little impact on the subjects and have a strong prospect for cooperative research. The Q value and S value in this clustering are relatively large, [Modularity (Q) assesses the strength of links within the community as compared to the connections outside the community, and is given by. It ranges from −1 to 1, where a higher value indicates a better partition. Moreover, a modularity of 0 indicates that the community structure established is indifferent to a randomly established partition (Srinivas and Rajendran, 2019). Given an item, an average silhouette width (ASW) close to 1 suggests a good clustering result, a value close to 0 suggests that it may belong to two clusters, and a value close to −1 suggests a wrong membership (Ping et al., 2017)] indicating that the clustering effect is ideal and modularity is remarkable, and the results are reasonable and can be used for analysis. Keyword timeline map and burst analysis show that since 2013, the research on MGBA in AD has been focusing on microbiota, and different hotspot bursts have appeared with the development of new theories and technologies.

### The microbiota is one of the most important concerns of MGBA in AD

There is evidence that microbiota changes in AD precede pathological changes and behavioral changes in the brain (Frausto et al., 2021), which means that detection or intervention of the microbiota holds promise for early diagnosis and treatment of AD.

#### Studies of microbiota in AD are diverse

The gut microbiota plays such an important role in the nervous system that it has been likened to The Second Brain (Ochoa-Reparaz and Kasper, 2016). The gut microbiota and the enteric neuroimmune system not only interact in neurodegenerative diseases, especially in AD, but can also be discussed in conjunction with obesity, type 2 diabetes, etc. (Naseer et al., 2014; Pellegrini et al., 2018). In terms of metabolomics, epigenetic molecular changes, etc., there is crosstalk between the gut microbiota and the brain in neurodegenerative diseases, especially AD (Luan et al., 2019; Nagu et al., 2021). In regard to the microbiota, it is natural to think of antibiotics for alleviation, and some studies illustrate the link between antibiotics, gut microbiota, and AD (Angelucci et al., 2019), and antibiotics can make “sterile” AD model mice (Wang et al., 2021). TCM treatments also play a role in AD through gut microbiota such as ginkgolide B, Nano-Honokiol (Liu J. M. et al., 2021; Qu et al., 2022; Sun et al., 2022).

#### The microbiota and nutrition are closely related

Lack of proper nutrition for microbiota is a major factor underpinning dysfunctional microbiota, dysbiosis, chronically elevated inflammation, and the production and leakage of endotoxins through the various tissue barriers (Bengmark, 2013). Consistent with conventional nutrition, chemicals in dietary plants influence gut microbiota (Wang et al., 2022), and calorie restriction slows age-related microbiome changes in AD (Cox et al., 2019). Studies also show that vitamin A deficiency exacerbates gut dysbiosis and cognitive deficits (Chen et al., 2021).

### Treatment is at the heart of AD research

Research on AD treatment has been evolving in a variety of ways, such as anti-etiological immunotherapy, gene therapy, anti-inflammatory therapy, anti-amyloid and anti-tau treatment strategies, symptomatic treatments such as glutamatergic, cholinergic-based therapy, and combining cutting-edge areas of artificial intelligence. The emerging probiotic treatments, nutraceuticals and dietary interventions can all be closely linked to MGBA (Ghosh et al., 2020; Khan et al., 2020). As mentioned above, the microbiota’s own structure and the fatty acids, neurotransmitters and other metabolites it produces are associated with AD and other neurological diseases through mechanisms such as inflammation. Therefore, microbiota-based treatments such as fecal transplants and probiotic treatment can work together through multiple mechanisms, which means opportunities and challenges. Once a breakthrough is found, it means that there may be common treatment methods for multiple diseases, and the most ideal situation may just require a change in diet.

First, transplantation of gut microbiota derived from a mouse model of Alzheimer’s disease impairs memory function and neurogenesis in mice (Kim N. et al., 2021), Based on this, if there is a correlation between microbiota changes and AD, it is reasonable to speculate that improving the microbiota may become an effective treatment for AD. Among the studies were microbiota transplants (fecal transplants), probiotics, and other chemotherapy treatments. For example, microbiota transplantation treatment can inhibit the activation of astrocytes around Aβ plaques (Wang et al., 2021), and shifting healthy bacterial populations reduces amyloid and tau pathology and more (Kim et al., 2020).

Probiotic treatment improves gut microbiota, a potential treatment option for Alzheimer’s disease (Ji and Shen, 2021). Additionally, probiotics modulate the microbiome-gut-brain axis, improve memory deficits, promote mental flexibility, and reduce stress (Yang et al., 2020; Kim C. S. et al., 2021), among them, Clostridium butyricum, Lactobacillus plantarum, and Bifidobacterium longum can attenuate neuroinflammation and reduce cognitive impairment in mice (Sun et al., 2020; Lee et al., 2021). Treatment with the compound probiotic SLAB51 affected the composition of the gut microbiota and its metabolites, thereby affecting the plasma concentrations of inflammatory cytokines and key metabolic hormones associated with neurodegenerative diseases (Bonfili et al., 2017).

In addition to microbiota transplantation and probiotics, the therapeutic effects of a variety of other drugs have also been extensively studied. For example, yeast beta-glucan modulates the gut microbiota and alleviates cognitive deficits through the influence of the insulin signaling system (Xu et al., 2020a,b). 27-Hydroxycholesterol (Wang et al., 2020), sodium oligomannate (Wang X. Y. et al., 2019), mannan oligosaccharide (Liu Q. et al., 2021; Liu S. et al., 2021), polyphenols (Shabbir et al., 2021) and fasudil (Yan et al., 2021), etc. have achieved good results in animal experiments. They improve cognition and behavior in mice by reshaping the gut microbiota and blood–brain barrier, attenuating inflammation and oxidative stress, and inhibiting Rho kinase.

### Pathology of microbiota and AD

In terms of AD pathology research, both the amyloid deposition theory and the tau pathology theory are widely recognized theories of AD pathogenesis, and gut microbiota plays a key role in amyloid production and pathogenesis in AD, affecting tauopathy in mouse models (Pistollato et al., 2016; Harach et al., 2017; Sun et al., 2019). The application of model mice in pathological mechanism research experiments is very important. Intestinal microbiota and AD-related studies have been intensively studied in mice such as TgCRND8 mice (Qu et al., 2022), SAMP8 mice (Zhan et al., 2018), APPPS1 mice (Li et al., 2020), and 5xFAD mice (Mezo et al., 2020).

### References bursts

After sorting out the context of the literature, the author found that several references burst out, which shows that they are very important and it is necessary to discuss them separately (Figure 10; Supplementary Figure 1). Harach et al. (2017) measured significant changes in fecal 16S rRNA in APP transgenic mouse models compared with wild-type mice. This study found that Aβ amyloid pathology was significantly reduced in germ-free APP transgenic mice while transplanting the microbiota of conventionally reared APP transgenic mice into germ-free APP mice increased brain Aβ pathology. In contrast, the microbiota of transplanted conventionally reared wild-type mice did not significantly increase brain Aβ (Harach et al., 2017). This study greatly advances the application of AD disease model mice in MGBA and AD research.

The inflammation theory has always been an important component of the pathological theory of AD, generally referring to inflammation in the central nervous system. The study by Cattaneo et al. (2017) linked the increase in intestinal pro-inflammatory microbiota taxon Escherichia/Shigella and reduction in the abundance of an anti-inflammatory taxon to peripheral inflammation and cognitive impairment and brain amyloidosis. The gut microbiota of AD patients may contribute to the exacerbation of AD pathology, with significantly reduced brain amyloid-β plaques and neurofibrillary tangles pathology in germ-free 3xTg mice. The gut microbiome of AD patients exacerbates AD pathology in germ-free 3xTg mice compared with specific pathogen-free mice (Chen et al., 2022). In addition to empirical research, the development of review research is also a key link in the development of a certain scientific research field. An extensive review by Jiang illustrates that research on the microbiota-gut-brain axis involves neural, immune, endocrine, and metabolic pathways, and the role of the gut microbiota in host cognition and AD pathogenesis have been identified through studies among germ-free animals, common animals, and fecal microbial transplant animals. In addition, bacteria in the gut microbiota can secrete large amounts of amyloid and lipopolysaccharides, and gut microbiota imbalances can also induce inflammation associated with the pathogenesis of obesity, type 2 diabetes, and AD (Jiang et al., 2017).

### Conclusion

To data, the author has conducted a preliminary analysis of 608 studies of MGBA in AD in the past 10 years based on the WOS core collection. Although the number of included articles was not large, which may limit the analysis, the number of research studies in this field is growing rapidly every year, and timely sorting of the literature is needed to further promote the development of this field. Based on the above analysis, the author found that there are many countries in the field of cooperation and the research on MGBA in AD has a certain foundation, but there are still shortcomings MGBA. For example, numbers of review articles and empirical research articles are almost equal, which shows that the research context in this field needs to be clarified continuously. This is a major problem in the current field. Our analysis also uncovered other limitations of the current study, such as the fact that while changes to the microbiota can be made in the short term, there is no conclusiveness about how long the altered microbiota can affect the brain, which may be a specific research direction. In addition, the mechanism of action of different gut microbiota constituents in AD is still unclear. Microbiota transplantation, probiotic therapy, and other chemotherapy methods have made little progress in the field of AD clinical treatment research, and the efficacy is not clear. Through this bibliometric analysis, researchers can timely control the development of this field, broaden research ideas, and then promote in-depth research on MGBA and AD mechanism.

## Data availability statement

The original contributions presented in the study are included in the article/Supplementary material, further inquiries can be directed to the corresponding author.

## Author contributions

Z-LL performed most of the experiments, screened the literature, analyzed experimental data, and drafted the manuscript. MW made some figures and reviewed the conclusions. H-TM also screened the literature and reviewed the manuscript. Y-HQ reviewed the manuscript again and approved the final draft. All authors contributed to the article and approved the submitted version.

## Funding

The authors sincerely thank the National Natural Science Foundation of China (No. 81571251, 81071035) and the Shaanxi Provincial Natural Science Basic Research Program (2019JZ-21) for funding this study. The funder had no role in any part of the design and implementation of manuscript editing and submission.

## Conflict of interest

The authors declare that the research was conducted in the absence of any commercial or financial relationships that could be construed as a potential conflict of interest.

## Publisher’s note

All claims expressed in this article are solely those of the authors and do not necessarily represent those of their affiliated organizations, or those of the publisher, the editors and the reviewers. Any product that may be evaluated in this article, or claim that may be made by its manufacturer, is not guaranteed or endorsed by the publisher.

## Abbreviations

MGBA: Microbiota-gut-brain axis
WOS: Web of Science database
AD: Alzheimer’s disease
MCI: Mild cognitive impairment
SCI: Science Citation Index
ASW: Average silhouette width
TCM: Traditional Chinese medicines
APP: Amyloid precursor protein
PS1: Presenilin 1
SAMP8: Senescence-accelerated mouse prone 8
