# Supplementary material for: Research trend of microbiota-gut-brain axis in Alzheimer’s disease based on CiteSpace (2012–2021): A bibliometrics analysis of 608 articles
Source: Front Aging Neurosci. 2022 Nov 22;14:1036120. doi: 10.3389/fnagi.2022.1036120 (PMC9724362; doi:10.3389/fnagi.2022.1036120)
Supplement: Supplementary file 1 [file Data_Sheet_1.docx]

Supplement Document 1: Mesh Terms and Search Strategy

Literature data were collected from the Science Citation Index (SCI) of the database Web of Science Core Dataset. For the sake of comprehensiveness, through Mesh Terms identifies the search subject term as (TS=(intestinal flora) OR TS=(Gastrointestinal Microbiomes) OR TS=(Microbiome, Gastrointestinal) OR TS=(Gut Microbiome) OR TS=(Gut Microbiomes) OR TS=(Microbiome, Gut) OR TS=(Gut Microflora) OR TS=(Gut Microbiota) OR TS=(Gut Microbiotas) OR TS=(Microbiota, Gut) OR TS=(Gastrointestinal Flora) OR TS=(Flora, Gastrointestinal) OR TS=(Gut Flora) OR TS=(Flora, Gut) OR TS=(Gastrointestinal Microbiota) OR TS=(Gastrointestinal Microbiotas) OR TS=(Microbiota, Gastrointestinal) OR TS=(Gastrointestinal Microbial Community) OR TS=(Gastrointestinal Microbial Communities) OR TS=(Microbial Community, Gastrointestinal) OR TS=(Gastrointestinal Microflora) OR TS=(Microflora, Gastrointestinal) OR TS=(Gastric Microbiome) OR TS=(Gastric Microbiomes) OR TS=(Microbiome, Gastric) OR TS=(Intestinal Microbiome) OR TS=(Intestinal Microbiomes) OR TS=(Microbiome, Intestinal) OR TS=(Intestinal Microbiota) OR TS=(Intestinal Microbiotas) OR TS=(Microbiota, Intestinal) OR TS=(Intestinal Microflora) OR TS=(Microflora, Intestinal) OR TS=(Intestinal Flora) OR TS=(Flora, Intestinal) OR TS=(Enteric Bacteria) OR TS=(Bacteria, Enteric)) AND (TS=(Alzheimer OR Alzheimer Dementia OR Alzheimer Dementias OR Dementia, Alzheimer OR Alzheimer's Disease OR Dementia, Senile OR Senile Dementia OR Dementia, Alzheimer Type OR Alzheimer Type Dementia OR Alzheimer-Type Dementia (ATD) OR Alzheimer Type Dementia (ATD) OR Dementia, Alzheimer-Type (ATD) OR Alzheimer Type Senile Dementia OR Primary Senile Degenerative Dementia OR Dementia, Primary Senile Degenerative OR Alzheimer Sclerosis OR Sclerosis, Alzheimer OR Alzheimer Syndrome OR Alzheimer's Diseases OR Alzheimer Diseases OR Alzheimers Diseases OR Senile Dementia, Alzheimer Type OR Acute Confusional Senile Dementia OR Senile Dementia, Acute Confusional OR Dementia, Presenile OR Presenile Dementia OR Alzheimer Disease, Late Onset OR Late Onset Alzheimer Disease OR Alzheimer's Disease, Focal Onset OR Focal Onset Alzheimer's Disease OR Familial Alzheimer Disease (FAD) OR Alzheimer Disease, Familial (FAD) OR Familial Alzheimer Diseases (FAD) OR Alzheimer Disease, Early Onset OR Early Onset Alzheimer Disease OR Presenile Alzheimer Dementia)) and Web of Science Core Collection (Database) and Articles or Review Articles or Editorial Materials (Document Types), Types of articles collected include “Articles or Review Articles or Editorial Materials”.
