# Supplementary figures and images for: Research trend of microbiota-gut-brain axis in Alzheimer’s disease based on CiteSpace (2012–2021): A bibliometrics analysis of 608 articles
Source: Front Aging Neurosci. 2022 Nov 22;14:1036120. doi: 10.3389/fnagi.2022.1036120 (PMC9724362; doi:10.3389/fnagi.2022.1036120)

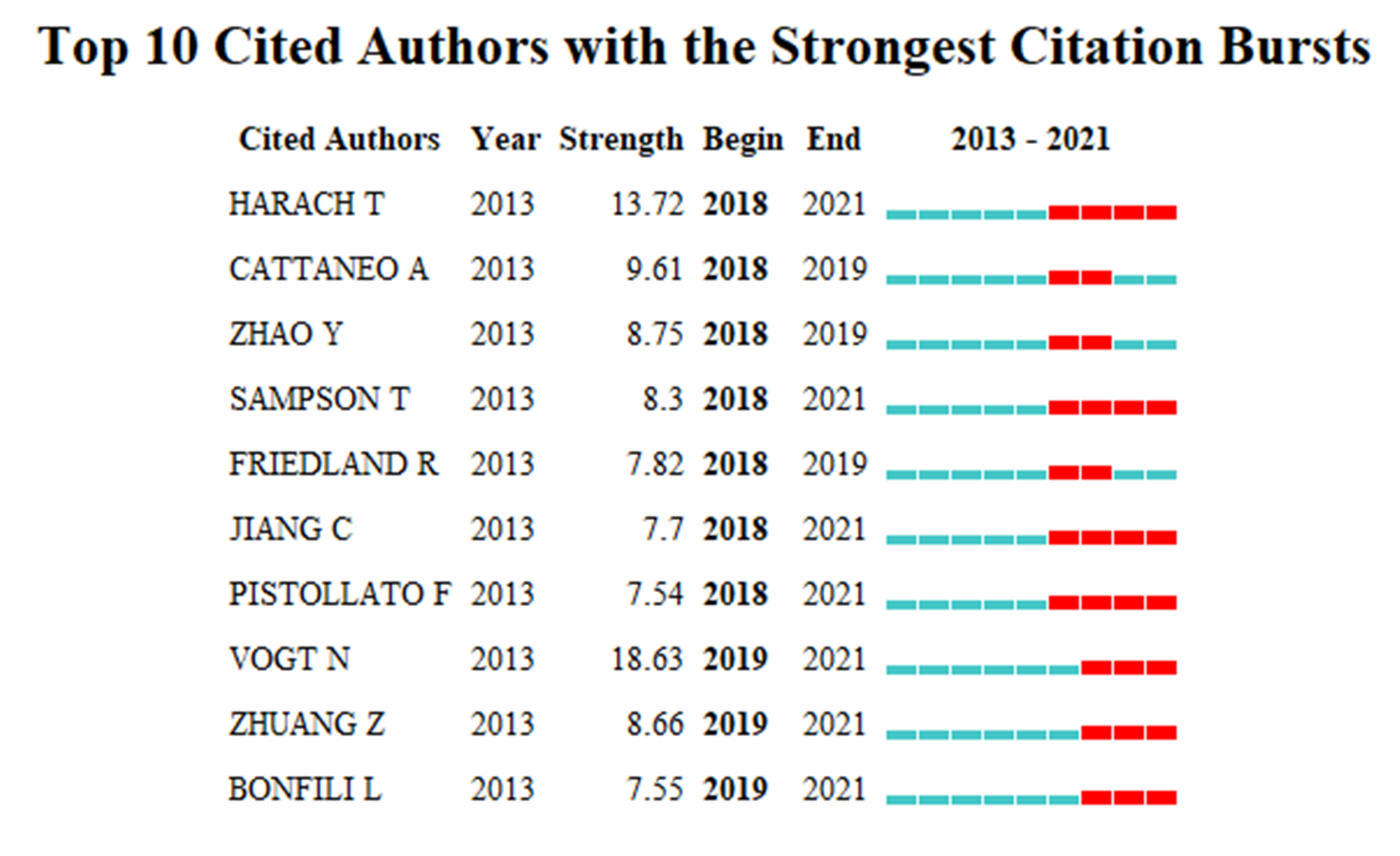

Supplement: Supplementary file 2 [file Image_1.TIF]
